# Effect of Phytoplankton Richness on Phytoplankton Biomass Is Weak Where the Distribution of Herbivores is Patchy

**DOI:** 10.1371/journal.pone.0156057

**Published:** 2016-05-19

**Authors:** Jerome J. Weis

**Affiliations:** Department of Ecology and Evolutionary Biology, Yale University, New Haven, Connecticut, United States of America; Evolutionary Biology Centre (EBC), Uppsala University, SWEDEN

## Abstract

Positive effects of competitor species richness on competitor productivity can be more pronounced at a scale that includes heterogeneity in ‘bottom-up’ environmental factors, such as the supply of limiting nutrients. The effect of species richness is not well understood in landscapes where variation in ‘top-down’ factors, such as the abundance of predators or herbivores, has a strong influence competitor communities. I asked how phytoplankton species richness directly influenced standing phytoplankton biomass in replicate microcosm regions where one patch had a population of herbivores (*Daphnia pulicaria)* and one patch did not have herbivores. The effect of phytoplankton richness on standing phytoplankton biomass was positive but weak and not statistically significant at this regional scale. Among no-*Daphnia* patches, there was a significant positive effect of phytoplankton richness that resulted from positive selection effects for two dominant and productive species in polycultures. Among with-*Daphnia* patches there was not a significant effect of phytoplankton richness. The same two species dominated species-rich polycultures in no- and with-*Daphnia* patches but both species were relatively vulnerable to consumption by *Daphnia*. Consistent with previous studies, this experiment shows a measurable positive influence of primary producer richness on biomass when herbivores were absent. It also shows that given the patchy distribution of herbivores at a regional scale, a regional positive effect was not detected.

## Introduction

The diversity of competing species influences the magnitude of ecosystem functions such as the uptake of limiting resources and the production of biomass in food webs [1–4]. Experiments have shown that competitor richness tends to have a positive effect on competitor biomass and productivity [3–5]. This positive effect is often linked to the more efficient uptake of resources in species-rich communities; either because species use resources in complementary ways under complex environmental conditions (positive complementarity effects) or because increased richness increases the likelihood of including highly productive and dominant species (positive selection effects)[6–8]. However, the strength and even the direction of the direct effects of competitor species richness can vary and are not well quantified in many realistic environments and food webs [2,9]. To date, most empirical assessments of the direct effects of competitor species richness come from experimental manipulations of species richness [10], where direct effects can be easily quantified [1]. Individual experimental units often lack important components of environmental heterogeneity that allow for realistic competitive interactions and niche partitioning among species [11,12]. Experiments that have simultaneously manipulated species richness and environmental heterogeneity, either within or among experimental units, have shown that positive effects of species richness on competitor productivity can be more pronounced at a scale that includes this heterogeneity [13–16]. These experiments have addressed environmental heterogeneity in ‘bottom-up’ environmental factors such as the balance of limiting resources or the frequency of disturbance. The effect of species richness is not well understood in landscapes where variation in ‘top-down’ factors, such as the abundance of predators, has a strong influence on the richness, composition, and productivity of competitor communities at lower trophic levels (for example [17,18]).

In landscapes where populations at an upper ‘predator’ trophic level have a patchy distribution in time or space, competing ‘prey’ species that vary in their vulnerability to predation may partition resources and coexist primarily at a regional scale [19]. In the absence or low abundance of predators highly productive prey species may dominate species-rich prey communities. The increased incidence of these productive and dominant prey species in species-rich communities can result in a positive selection effect of prey species richness in the absence of predators [7,20]. In the presence or high abundance of predators highly resistant prey species may dominate species-rich prey communities. The increased incidence of these resistant and dominant prey species in species-rich communities can result in a positive selection effect of prey species richness in the presence of predators [20,21]. If different prey species drive positive selection effects in the presence and absence of predators, perhaps due to a tradeoff between productivity and resistance to predation, then in a region where the presence of predators is patchy there may be a positive complementarity effect of regional species richness [20]. At this scale total regional prey biomass in species-rich assemblages may be higher than the biomass of any single prey species grown alone in monoculture; a phenomenon known as transgressive overyielding [22] which is associated with positive complementarity effects [4,23]. However, the strength and the direction of the direct effects of prey species richness on prey productivity can vary substantially in the presence of predators [21,24–27] and in some landscapes, the presence of predators and herbivores may result in negative or roughly neutral direct effects of prey richness on prey biomass at local and regional scales [20]. Relatively vulnerable prey species may still dominate species-rich prey assemblages in the presence of predators due to some other competitive advantage. The increased incidence of these vulnerable but dominant prey species in species-rich communities can result in a negative or roughly neutral relationship between prey species richness and prey biomass in the presence of predators [20,21]; what can be thought of as a negative selection effect of species richness [28–30].

If the presence and distribution of predators and herbivores can mechanistically alter the strength and direction of the direct influence of prey species richness on prey biomass then the effects of these upper trophic level consumers should be considered in food webs where productivity at lower trophic levels is under top-down control [2]. Here I ask how phytoplankton species richness influences phytoplankton biomass when the distribution of zooplankton herbivores is patchy. Experiments have shown that, on average, phytoplankton richness tends to have a positive effect on standing phytoplankton biomass in the absence of herbivores, but the effect of phytoplankton richness in the presence of herbivores is less clear [31]. Temperate lake phytoplankton species vary substantially in vulnerability to herbivory. Phytoplankton species also compete at least in part through a tradeoff between the ability to take up limiting resources and the ability to resist herbivory, generally associated with variation in cell and colony size [32,33]. This ‘competition-resistance’ tradeoff may allow phytoplankton species to coexist and partition resources at a regional scale where the influence of herbivores is patchy. For instance, in Coastal New England lakes variation in the density of herbivorous zooplankton among lakes, driven primarily by the patchy distribution of zooplanktivorous fish, results in variation in phytoplankton biomass and community composition [34–36]. If herbivory by zooplankton influences phytoplankton communities within lakes and at a more regional scale among lakes then studies that incorporate consumption by zooplankton at both of these scales may more accurately represent the effect of phytoplankton species richness in these ecosystems.

## Methods

### Plankton

I manipulated the richness of an assemblage of five phytoplankton species common in New England lake communities. I isolated cultures directly from Southern Connecticut lakes or purchased them from the University of Texas at Austin Culture Collection (UTEX). This group included three diatom species (division Bacillariophyta); *Asterionella* sp. (Pattagansett Lake, Connecticut, USA), *Cyclotella quillensis* (UTEX—LB FD142), and *Navicula* sp. (Bride Lake, Connecticut, USA), and two Chlorophyceaen green species; *Chlamydomonas moewusii* (UTEX—1053), and *Mougeotia* sp. (Rogers Lake, Connecticut, USA). I chose these species for three reasons; (1) they represent a diversity of functional traits, including growth form and cell size, associated with competitive ability and resistance to herbivory (Table 1)[32,33], (2) they represent the dominant phylogenetic groups and growth forms of phytoplankton communities in Southern Connecticut lakes [34,35], and (3) they grew well under my experimental conditions. I also manipulated the presence or absence of a single crustacean herbivore *Daphnia pulicaria*. *Daphnia* populations originated from one individual hatched from a sediment ephippia collected at Linsley Pond; a well-studied seasonally stratified lake in Southern Connecticut, USA [17,35,36]. Prior to the experiment I raised *Daphnia* populations in COMBO growth media [37] on a population of *Navicula* as the food source. All plankton used in this study are not endangered nor protected species. Special permission was not required to collect water nor sediment samples. Permission was granted by Yale University to sample at Linsley Pond and by the Connecticut Department of Correction to sample at Bride Lake. Pattagansett Lake is open to public access.

**Table 1:** I used five freshwater phytoplankton species common in North American lakes in this experiment. Species represented two major phylogenetic divisions, several common growth forms, and varied in their ability to remain in the water column. Under the experimental conditions species varied in average cell biomass and edibility to the zooplankton herbivore (*Daphnia pulicaria*).

Species	Division	Growth Form	Water Column	Cell Biomass (ng)	Edibility
*Asterionella sp*.	Bacillariophyta	Solitary/Colonial	Sink	0.21	Yes
*Cyclotella quillensis*	Bacillariophyta	Solitary	Sink	0.24	Yes
*Navicula sp*.	Bacillariophyta	Solitary	Sink	0.16	Yes
*Chlamydomonas moewusii*	Chlorophyta	Solitary	Motile	0.09	Yes
*Mougeotia sp*.	Chlorophyta	Fillamentous	Float	0.72	No

### Experimental design

I used clear jars filled with 400ml of autoclaved COMBO growth media and capped with sterile cotton stoppers as experimental units. I manipulated phytoplankton richness (1,3, or 5 species) across pairs (regions) of two adjacent jars. Both jars within a region were seeded with the same initial phytoplankton community and one of the two jars, randomly designated the with-*Daphnia* patch, was seeded with an initial *Daphnia* population. This design allowed me to analyze summed phytoplankton biomass at a regional scale consisting of one no-*Daphnia* patch and one with-*Daphnia* patch following the approach used by Weis and Vasseur [20]. It also allows me to evaluate effects of phytoplankton richness mechanistically within no- and with-*Daphnia* patches individually. I included four replicate regions of each 1-species monoculture (20 regions), eleven regions of 3-species polycultures including one replicate of each possible 3-species combination and one additional replicate of the 3-diatom combination, and five replicate regions of the 5-species polyculture (total of 36 regions or 72 jars).

On the start day of the experiment (DOE 0) I estimated stock culture cell density of each of the phytoplankton species from cell counts using a hemocytometer. I seeded each jar with 10^6^ phytoplankton cells (2500 cells/mL). To keep initial phytoplankton cell density constant across phytoplankton richness treatments I reduced the initial density of each species as richness increased; what is commonly referred to as a substitutive design across species richness treatments. To seed *Daphnia* populations I rinsed gravid adult *Daphnia* four times in autoclaved COMBO and left each in sterile COMBO over-night in complete darkness on DOE -1,0, and, 1. Each following morning I rinsed newly born juveniles once in sterile COMBO. On DOE 0 and DOE 1 I seeded a single one-day-old juvenile *Daphnia* in the designated with-*Daphnia* patch within each region (total initial population of two juveniles). To minimize the effects of early stochastic mortality in these seed populations I replaced dead *Daphnia* with one-day-old juveniles on DOE 1 and 2. Between DOE 0 and 1, eight of 36 juvenile *Daphnia* died and were replaced. Between DOE 1 and 2, seven of 72 juvenile *Daphnia* died and were replaced. This early mortality was dispersed across phytoplankton richness treatments. For contrast, between DOE 2 and 4 four of 72 *Daphnia* died and each of these four was in a phytoplankton monoculture. I did not replace these or any other *Daphnia* that died after DOE 2.

I conducted the experiment in a growth chamber on a 15:9 hour light:dark cycle at 20°C. I placed regions (pairs of jars touching front-to-back) on three evenly spaced shelves within the growth chamber in a 3x4 arrangement randomized across treatments. I lit each shelve individually with two white fluorescent light bulbs hung approximately 0.2M above the jars on opposite ends of the growth chamber and lined each shelve floor with white plastic. Every morning I swirled each jar by hand to maintain phytoplankton in the water column.

The experiment ran for three weeks from April 1^st^ (DOE 0) to April 23^rd^ 2013 (DOE 22). In general, across phytoplankton richness treatments, *Daphnia* and phytoplankton populations grew according to the first portion of a predator-prey cycle. Early in the experiment, when *Daphnia* populations were small, *Daphnia* did not appear to have a direct consumptive effect on phytoplankton biomass. Later in the experiment, as *Daphnia* and phytoplankton populations grew, *Daphnia* reduced phytoplankton biomass in with-*Daphnia* patches relative to no-*Daphnia* patches, but phytoplankton biomass was still high relative to the initial densities. Finally, as *Daphnia* populations continued to increase, consumption of phytoplankton by *Daphnia* reduced phytoplankton biomass to very low ‘clear-water’ levels in with-*Daphnia* patches. The transition from relatively high to very low phytoplankton biomass often occurred quickly (< 24 hours). In the with-*Daphnia* patches summary statistics of phytoplankton biomass from late in the experiment (DOE 19&22) often compare or include pre- and post-clear-water communities. I ended the experiment when with-*Daphnia* patches in the 5-species polycultures began reaching this clear-water phase.

A with-*Daphnia* patch within one of the 5-species polyculture replicates largely failed to grow. Only one of the two original juvenile *Daphnia* survived through the experiment and neither individual reproduced. In contrast, Daphnia populations increased in all other treatments and replicates where one or more edible phytoplankton species was present. Also in contrast to all other experimental units, this failed replicate became visibly dominated by heterotrophic bacteria. I excluded this failed replicate region (both jars) from all analyses. My analyses and conclusions are sensitive to including this with-*Daphnia* replicate since it did not have a viable *Daphnia* population. I also excluded one of the two 3-diatom polyculture replicates, randomly chosen, from my statistical analyses so that a single replicate of each 3-species combination was represented. My results and conclusions are not sensitive to which 3-diatom polyculture is excluded. Given these eliminations, my statistical analyses included four replicates of each 1-species monoculture, ten 3-species polycultures, and four replicates of the 5-species polyculture.

### Sampling

To estimate phytoplankton biomass I sampled the experiment every 3 days starting on DOE 4 (DOE 4,7,10,13,16,19, and 22). I removed 1mL subsamples from each jar and preserved these subsamples in diluted Lugol’s solution. I counted cell density from preserved samples using a hemocytometer at 200x magnification under a light microscope. Depending on the density of cells in each sample, I varied the grid area counted per sample until I had counted a minimum of approximately 400 cells per species or had counted four 9mm^2^ grids on the hemocytometer (3.6μL of the subsample). Following these cell density estimates, I measured linear dimensions of phytoplankton species from digital images using the software package Image J (v.1.6.0, National Institutes of Health, USA) and estimated cell biovolume for each species from the geometric equations outlined by Hillebrand [38]. I assumed that phytoplankton cell size may have varied across dates based on the availability of limiting resources. On each sampling date, I measured one haphazardly chosen cell of each species from each jar (~30 cells per species per date) to estimate the average biovolume of each species on that date. I then multiplied cell densities by average biovolume to estimate biovolume density of each species and converted biovolume density to biomass density assuming a specific gravity of 1.0.

I counted *Daphnia* visually in each jar every 3 days over a fluorescent light box. This counting method was reliable through DOE 10, when populations were still limited to less than 10 *Daphnia* in each jar. The live counts became unreliable as *Daphnia* populations increased so I only used these early live counts to confirm the survival of *Daphnia* populations. At the end of the experiment, DOE 22, after sampling phytoplankton I filtered the entire *Daphnia* population of each experimental unit through 80μ mesh and preserved *Daphnia* in 70% ethanol. I counted preserved *Daphnia* under a dissecting microscope. I also measured the length of 50 haphazardly chosen individuals, or of all individuals in jars with less than 50, with an ocular micrometer and used McCauley’s [39] length/biomass regression to estimate average *Daphnia* biomass. I then multiplied the average *Daphnia* biomass by the total number of *Daphnia* to estimate final *Daphnia* biomass per jar.

### Analysis

I conducted all statistical analyses and calculated response metrics in the software package R (v3.1.1, R Core Team 2014, Vienna Austria).

I first tested for a significant influence of phytoplankton richness on total regional phytoplankton biomass (summed no- and with-*Daphnia* patches, *log*_*e*_ transformed) using a linear mixed effect model (α = 0.05) from the nlme package in R [40]. I included fixed effects of phytoplankton species richness, time (DOE), the interaction of richness and time, and a random effect of experimental unit identity (region identity in the summed region analysis, and jar identity in the no- and with-*Daphnia* patch analysis) to account for the repeated-measures nature of the data. The number of experimental units and the variation in community composition differed across levels of species richness so I allowed different variance at each level of richness in the models. I also report similar mixed effects models testing for significant influences of phytoplankton richness in no- and with-*Daphnia* patches separately.

I analyzed the influence of phytoplankton richness in more detail among regions and within no- and with-*Daphnia* patches by comparing the phytoplankton biomass of 3- and 5-species polycultures (*P*) to average monoculture biomass $\left(\bar{M}\right)$ using a yield response metric (*D*)(Eq 1).

$$D=\frac{P-\bar{M}}{\bar{M}}$$ (1)

Given the substitutive design of the species richness treatments, this metric *D* is similar to Loreau’s [41] metric *D*_*T*_. Here a positive *D* indicates that polycultures yielded more biomass than the average monoculture and a negative *D* indicates polycultures yielded less biomass. I used a linear mixed effect model to ask if *D* differed significantly between the no- and with-*Daphnia* patches. I included fixed effects of time (DOE), phytoplankton richness as a factor (3 or 5 species), *Daphnia* presence, and the interactions of all three terms. I included a random effect of patch (jar) identity and allowed different variance at the two levels of species richness.

I quantified the effect of *Daphnia* on phytoplankton biomass across sampling dates and treatments using an effect size metric (*h*) that compares phytoplankton biomass in the no-*Daphnia* treatment (*N*) to biomass in the with-*Daphnia* treatment (*W*) within the paired two-patch regions (Eq 2).

$$h=\frac{W-N}{N}$$ (2)

Here a negative value of *h* indicates a negative effect (i.e. a consumptive effect) of *Daphnia* on phytoplankton biomass. I also quantified average biomass proportion of each species in 5-species polycultures through time and calculated Shannon’s Diversity (*H*) and evenness (*J*) through the experiment [42].

I tested for a significant effect of phytoplankton richness on *Daphnia* biomass in the with-*Daphnia* treatment on the final day of the experiment (DOE 22) using a generalized least squares linear model, also from the nlme package in R. I allowed different variance at each level of richness in this model as well.

## Results

### Phytoplankton richness on phytoplankton biomass

**Fig 1:**
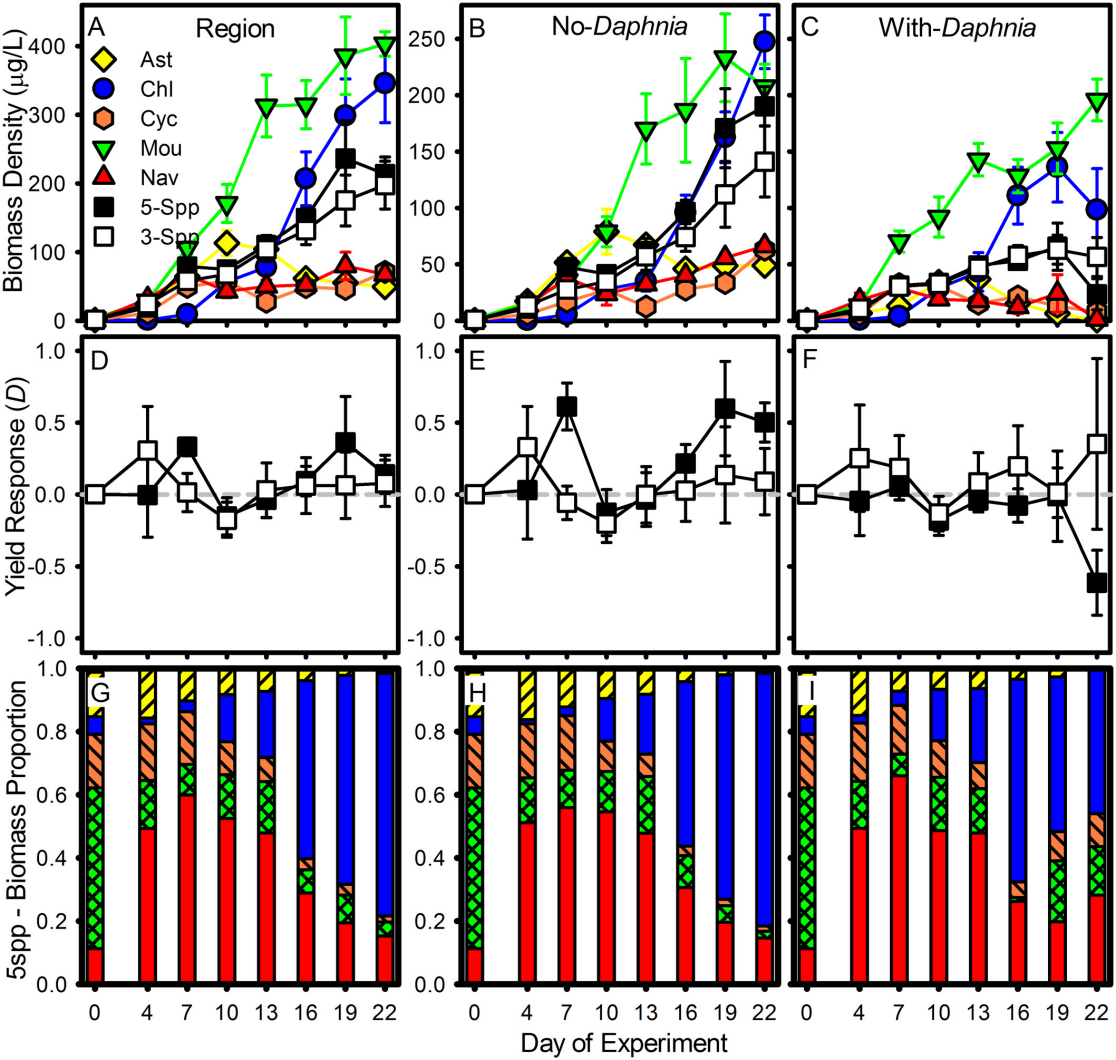
Phytoplankton Average Biomass and Yield Response Through Time. Top panels show average phytoplankton biomass density (± standard error) by phytoplankton species richness and monoculture treatments at the regional scale where biomass is summed across patches (A), in no-*Daphnia* patches (B), and in with-*Daphnia* patches (C). Colored shapes show phytoplankton monocultures; yellow diamonds—Asterionella (Ast), blue circles—Chlamydomonas (Chl), orange hexagons—Cyclotella (Cyc), green upside-down triangles—Mougeotia (Mou), red triangles—Navicula (Nav). Squares show species-rich polycultures; white squares– 3-species polycultures (3-Spp), black squares– 5-species polycultures (5-Spp). Middle panels show average yield response metric (D ± standard error) in the 3-species (white squares) and 5-species polycultures (black squares) at the regional scale (D), in no-*Daphnia* patches (E), and in with-*Daphnia* patches (F). Bottom panels show average percent composition by biomass of each phytoplankton species in the 5-species polycultures across sampling dates at the regional scale (G), in no-*Daphnia* patches (H), and in with-*Daphnia* patches (I).

Phytoplankton biomass increased by up to three orders of magnitude over the three-week study (Fig 1A, 1B, and 1C) and linear mixed effect models showed a highly significant positive effect of time on phytoplankton biomass at the regional scale and within the no- and with-*Daphnia* patches (Table 2). Phytoplankton species richness had a positive effect on total phytoplankton biomass among regions but this effect was not statistically significant (Fig 1A). Phytoplankton species richness did have a significant positive effect on species biomass among no-*Daphnia* patches (Fig 1B). Phytoplankton species richness did not have a significant effect on total phytoplankton biomass among with-*Daphnia* patches (Fig 1C).

**Table 2:** ANOVA results of linear mixed effect models of total phytoplankton biomass as fixed effects of time and phytoplankton species richness at the regional scale and within the no- and with-*Daphnia* patches.

	Region			No-*Daphnia*		With-*Daphnia*	
	DF	F	p	F	p	F	p
Time (DOE)	**1/236**	**455.08**	**<0.001**	**553.88**	**<0.001**	**105.61**	**<0.001**
Richness (R)	1/32	3.69	0.064	**4.50**	**0.042**	1.88	0.179
DOE:R	1/236	0.10	0.755	0.02	0.898	0.12	0.724

Across *Daphnia* treatments and sampling dates, average phytoplankton biomass in the 3- and 5-species polycultures did not exceed the average biomass of the most productive species in monoculture (Fig 1A, 1B, and 1C), indicating a lack of transgressive overyielding of polycultures in this experiment.

### Phytoplankton overyielding in polyculture

Average *D* of the 3-species polycultures was roughly equivalent to zero across sampling dates at the regional scale and within no- and with-*Daphnia* patches, indicating that the biomass of the 3-species polycultures was roughly similar to average monoculture biomass (Fig 1D, 1E, and 1F). At the regional scale, average *D* in the 5-species polycultures was positive on DOE 7 and DOE 19 (Fig 1D). In the no-*Daphnia* patches, average *D* in 5-species polycultures was positive on four of the seven sampling dates (Fig 1E), suggesting that overyielding in the 5-species polycultures drove the statistically significant positive influence of species richness on phytoplankton biomass detected by the analysis in the no-*Daphnia* patches (Fig 1B, Table 2). In the with-*Daphnia* patches, average D in the 5-species polycultures was negative on DOE 22 (Fig 1C).

Average *D* in no- and with-*Daphnia* patches did not differ significantly as a function of sampling date, richness (3 or 5), nor the presence of *Daphnia* (Table 3). Average *D* did differ significantly as a function of the full interaction of time, phytoplankton species richness, and the presence of *Daphnia* indicating that yield of species polycultures relative to monocultures did differ mechanistically between no- and with-*Daphnia* patches.

**Table 3:** ANOVA results of the linear mixed effect model of the polyculture yield metric (*D*) as a function of time, phytoplankton species richness, and the presence of *Daphnia*.

	DF	F	p
Time (DOE)	1/192	0.21	0.312
Richness (R)	1/24	0.03	0.650
*Daphnia*	1/24	0.28	0.868
DOE:R	1/192	0.02	0.599
DOE:*Daph*	1/192	3.35	0.069
R:*Daph*	1/24	2.11	0.159
DOE:R:*Daph*	1/192	**4.00**	**0.047**

### Phytoplankton biomass response to *Daphnia*

**Fig 2:**
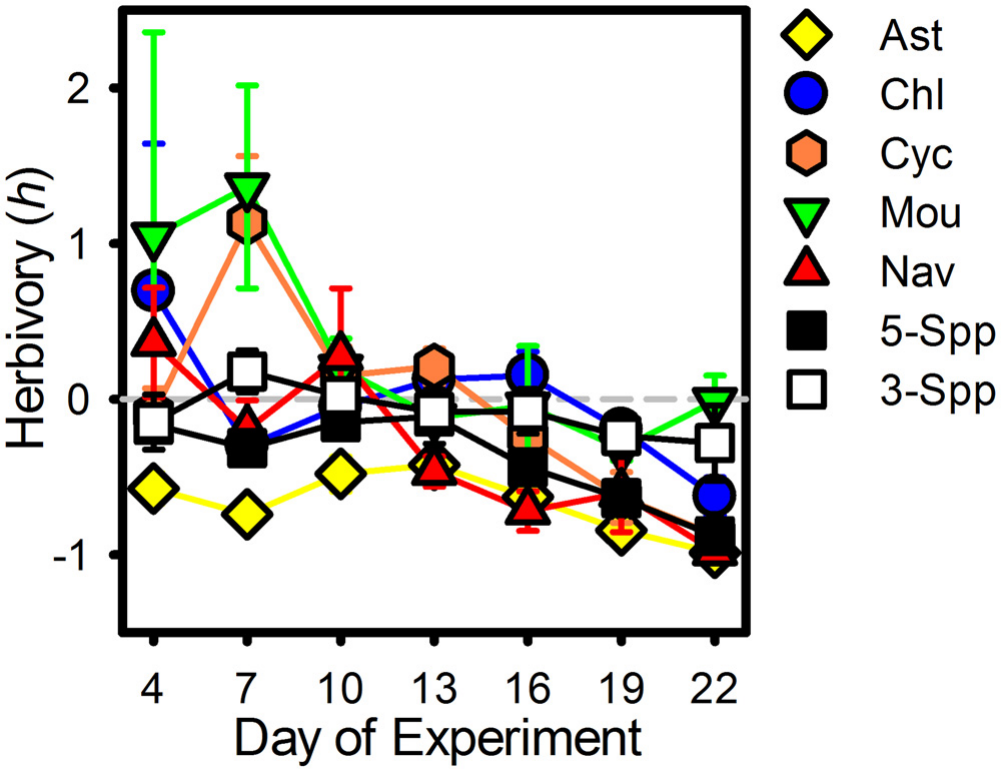
Phytoplankton Biomass Response to *Daphnia*. Average response to *Daphnia* within regions (*h*, ± standard error) across sampling dates is shown by phytoplankton treatments. Colored shapes show phytoplankton monocultures; yellow diamonds–*Asterionella* (Ast), blue circles–*Chlamydomonas* (Chl), orange hexagons–*Cyclotella* (Cyc), green upside-down triangles–*Mougeotia* (Mou), red triangles–*Navicula* (Nav). Squares show species-rich polycultures; white squares– 3-species polycultures (3-Spp), black squares– 5-species polycultures (5-Spp).

Early in the experiment, DOE 4 and 7, the average monoculture biomass of four of the five species was higher in with-*Daphnia* patches than no-*Daphnia* patches (positive *h* in Fig 2), but the variability around these averages was high. After DOE 10 in *Mougeotia* monocultures, when all *Daphnia* had died, biomass was roughly similar in no- and with-*Daphnia* patches (*h* near 0 in Fig 2). The onset of negative effects of *Daphnia* on average monoculture biomass (negative *h* in Fig 2) in the four ‘edible’ phytoplankton species varied. In monocultures of *Asterionella*, *Daphnia* had a negative effect on phytoplankton biomass through the course of the experiment. In monocultures of *Navicula* and *Cyclotella*, with-*Daphnia* biomass was lower than no-*Daphnia* biomass starting in the middle of the experiment (DOE 13 or 16). In monocultures of *Chlamydomonas*, with-Daphnia biomass was lower only late in the experiment (DOE 19&22). By the end of the experiment (DOE 22) *Daphnia* populations had reduced phytoplankton biomass to low ‘clear-water’ levels (<10μg/L approximately) in all diatom monoculture replicates and two of the four *Chlamydomonas* monoculture replicates.

All of the 3- and 5-species polyculture replicates in the with-*Daphnia* patches supported *Daphnia* populations. In 5-species polycultures, *Daphnia* populations had a negative influence on average total phytoplankton biomass, relative to the no-*Daphnia* treatment, through the course of the experiment (Fig 2). On DOE 22 *Daphnia* populations had reduced phytoplankton biomass to less than 5μg/L in two of the four 5-species polyculture replicates and three of the ten 3-species polycultures.

### Phytoplankton community composition in polyculture

Phytoplankton diversity (*H*) and evenness (*J*) tended to decrease through the experiment, particularly in no-*Daphnia* patches (S1 Fig). At the regional scale and in the no-*Daphnia* patches, 5-species polycultures were dominated early in the experiment by diatoms, particularly *Navicula*, and dominated late in the experiment by *Chlamydomona*s (Fig 1G and 1H). In the with-*Daphnia* treatment, 5-species polycultures were also dominated early by diatoms, particularly *Navicula*, and dominated late by *Chlamydomonas* (Fig 1I). Across sampling dates *Mougeotia* never accounted for more than 20% of the total biomass in the 5-species polycultures (Fig 1G, 1H, and 1I).

### Selection effects of phytoplankton richness on phytoplankton biomass

**Fig 3:**
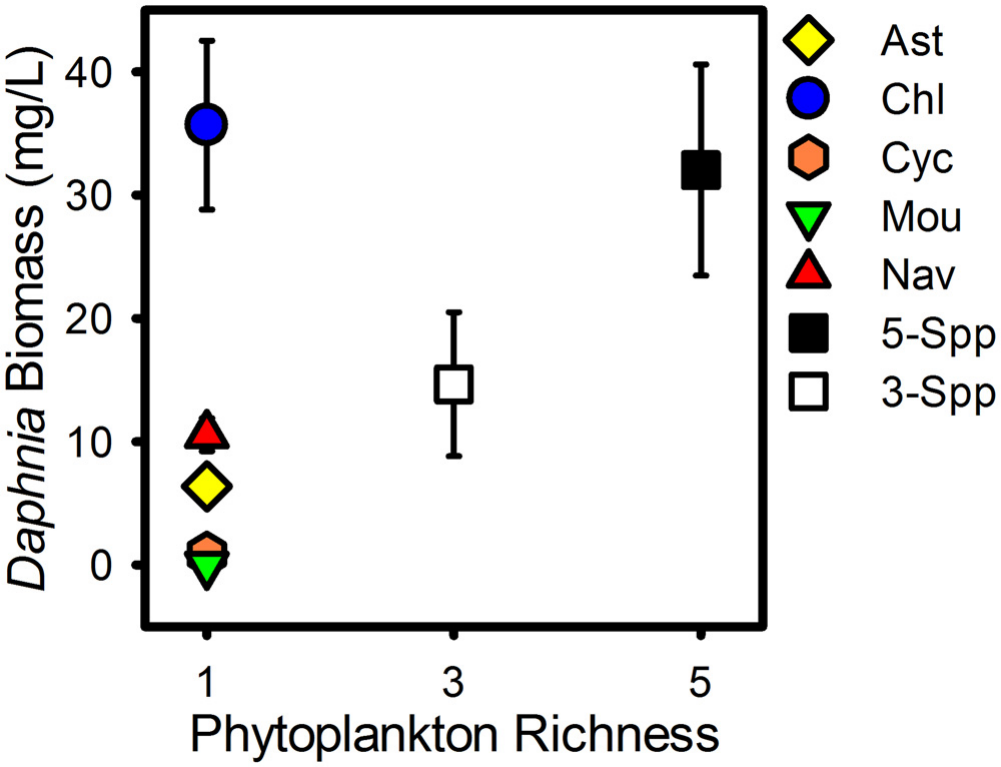
Final *Daphnia* Biomass. Average *Daphnia* biomass on the final day of the experiment (DOE 22) by phytoplankton treatment. Colored shapes show phytoplankton monocultures; yellow diamonds–*Asterionella* (Ast), blue circles–*Chlamydomonas* (Chl), orange hexagons–*Cyclotella* (Cyc), green upside-down triangles–*Mougeotia* (Mou), red triangles–*Navicula* (Nav). Squares show species-rich polycultures; white squares– 3-species polycultures (3-Spp), black squares– 5-species polycultures (5-Spp).

At time periods where there was distinct overyielding of 5-species polycultures, mostly in the no-Daphnia patches, 5-species polycultures were dominated by highly productive species, strongly suggesting that positive selection effects were more influential that positive complementarity effects. In the no-*Daphnia* patches, the significant positive effect of species richness can be explained largely by the increased likelihood of including two productive and dominant species, *Navicula* and *Chlamydomonas*, in species-rich polycultures; known as a positive selection effect of species richness. Early in the experiment, DOE 7, the fast-growing diatom species *Navicula* had relatively high average biomass in monoculture (38 μg/L)(Fig 1B) and dominated 5-species polycultures (Fig 3E). In contrast, by DOE 10 *Asterionella* and *Mougeotia* were the highest producing species in monoculture (Fig 1B) but neither of these species was dominant and highly productive in 5-species polycultures (Fig 3E). Between DOE 10 and DOE 19, polycultures did not yield more biomass than average monocultures (Fig 1E). By the end of the experiment *Chlamydomonas* had the highest average biomass in monoculture (247 μg/L) (Fig 1B). On DOE 22 *Chlamydomonas* dominated 5-species polycultures (Fig 3E) and these polycultures produced more biomass than the average monoculture (Fig 1E).

In the with-*Daphnia* patches I did not observe a positive selection effect driven by the phytoplankton species resistant to consumption by *Daphnia*, *Mougeotia*. Instead, 5-species polycultures were more likely to include the two dominant species, *Navicula* and *Chlamydomonas*, which where were both vulnerable to herbivory and therefore not nearly as productive as Mougeotia in with-Daphnia monocultures; what can be thought of as a negative selection effect of species richness [28–30]. In the with-*Daphnia* treatment *Mougeotia* had the highest average monoculture biomass among species by DOE 7, reaching 196 μg/L on DOE 22 (Fig 1C). However, *Mougeotia* was never the competitive dominant in 5-species polycultures. *Navicula* and *Chlamydomonas* dominated 5-species polycultures, but both of these species were vulnerable to consumption by *Daphnia* (Fig 2) and produced less biomass in monoculture than *Mougeotia* (Fig 1C). Species-rich polycultures were more likely to include these two vulnerable but dominant species leading to high overall phytoplankton consumption by *Daphnia* in polycultures (Fig 2).

At the regional scale positive selection effects driven by *Navicula* and *Chlamydomonas* in no-Daphnia patches and negative selection effects driven by the same two species in with-*Daphnia* patches balanced to result in the weaker and non-significant positive effect of species richness. These two species dominated polyculture biomass (Fig 3D) but neither species was the most productive in monoculture (Fig 1A). Instead, by DOE 7 monocultures of *Mougeotia* had the highest average monoculture biomass, reaching over 400 μg/L by DOE 22 (Fig 1A).

### Phytoplankton richness on *Daphnia* biomass

One DOE 22 there was a significant positive influence of phytoplankton species richness and *Daphnia* biomass (generalized least squares linear model; t = 4.80, df = 34, p<0.001). Average *Daphnia* biomass was greatest in *Chlamydomonas* monocultures (35mg/L) and lowest in monocultures of *Cyclotella* (0.9mg/L) and the inedible species *Mougeotia* (0mg/L)(Fig 3). Average Daphna biomass in the 5-species polycultures was 32mg/L.

## Discussion

In this experiment I did observe a statistically significant positive effect of phytoplankton richness within no-*Daphnia* patches driven by positive but temporally dynamic selection effects of two highly productive and dominant species. This result is mechanistically similar to the results of other experimental manipulations of phytoplankton richness in the absence of herbivores [25,28]. This result is also consistent more generally with experimental results that have collectively lead to the broad conclusion that competitor species richness tends to have a positive effect on competitor biomass [10,43]. However, I was unable to detect a strong positive effect of phytoplankton richness on phytoplankton biomass at a regional scale where there presence of *Daphnia* was patchy. Overall, the effect of regional phytoplankton richness on phytoplankton biomass was weak relative to changes in phytoplankton biomass over time and relative to the effect of herbivory in with-*Daphnia* patches (Fig 1A, 1B, and 1C, Table 2). Phytoplankton richness did not have a significant positive effect at the regional scale because there was not a strong positive effect of phytoplankton richness within with-*Daphnia* patches (Fig 1C and 1F). Negative selection effects driven by dominant but vulnerable species in with-*Daphnia* polycultures reduced the influence of any potential positive selection effect driven by resistant species. This result is consistent with other experiments that have failed to detect significant positive effects of primary producer species richness when herbivores are present [25,26]. This result is also consistent with theory that predicts negative or neutral effects of prey species richness when prey species do not show a strong tradeoff between resource uptake and resistant to predation [20].

There was a significant positive effect of phytoplankton species richness on average *Daphnia* biomass at the end of the experiment (Fig 3). It is possible that the positive relationship between phytoplankton species richness and *Daphnia* biomass resulted from the higher likelihood of including highly productive and highly edible phytoplankton species, particularly *Chlamydomonas*, in species-rich polycultures. Indeed, monocultures of *Chlamydomonas* produced higher average *Daphnia* biomass than 3- and 5-species polycultures. This phenomenon can be thought of as a positive selection effect of prey richness on predator biomass. More generally, this result is consistent with food chain theory that predicts higher standing biomass at the second trophic level in more productive two-trophic level food chains [44,45]. However the effect of prey species richness on predator productivity is not yet thoroughly characterized and cannot be consistently predicted from the number of trophic levels present. Previous manipulations of phytoplankton richness have reported a range of positive and negative influences of phytoplankton richness on zooplankton biomass [45–48] and there are a number of hypotheses proposed to explain this range of results (reviewed in [2,45,47]). Many of these hypotheses are based on the principle that prey species vary in vulnerability to predation. It may be possible to integrate some these hypotheses into a single more general understanding of the influences of prey species richness on predator productivity but this integration is beyond the scope of this study. In this experiment, which focused primarily in phytoplankton productivity, I was only able to collect a ‘snapshot’ of *Daphnia* biomass data from the final date of the experiment. *Daphnia* populations are known to follow predator-prey cycles in microcosms and natural systems [49].

Extending the conclusions of a 400ml microcosm study to lake phytoplankton communities requires consideration. Like all mesocosm studies the results of this lab experiment are limited to some extent to the environment and focal community of the study. Lake food webs are much more complex than the food web I address here and often support 50 or 100 species of phytoplankton [50]. It is possible that including more phytoplankton species in this study could have qualitatively changed the results. In general, microcosms are less complex environments than lakes and realistic variation in temperature, light, turbulence, and other physical factors may have changed my results. However, phytoplankton size and growth form affect the uptake of resources, sinking rates, and resistance to consumption by zooplankton in qualitatively similar ways in lakes and microcosms. One of the strengths of mesocosm experiments like this one is the ability to better understand the potential mechanisms behind direct effects of species richness in more realistic assemblages and environments, which can then be related to and tested for in natural environments. In temperate lakes, early in the growing season plankton densities are low and limiting nutrients are high in the photic zone; qualitatively similar starting conditions to the mesocosms studied here. As environmental conditions change through a growing season, the composition of phytoplankton communities changes [51,52]. Phytoplankton communities, including those of Coastal New England, are often dominated early in the growing season by non-motile diatoms which grow quickly when limiting nutrients are abundant [34,35]. Early in this experiment 5-species assemblages where dominated by non-motile diatoms, which grew quickly in the first week of the experiment when inorganic resources and light were presumably the least limiting to growth. Late in the season soft-bodied phytoplankton species including Chlorophycean green algae tend become more dominant in lakes [51,52]. Late in this experiment a Chlorophycean green species *Chlamydomonas*, a genus known to be dominant and influential in mesocosm experiments [28,45], dominated 5-species assemblages. This late dominance by *Chlamydomonas* in the 5-species assemblages may have resulted at least in part from variation in the mobility of our species assemblage. *Chlamydomonas* is a motile species and was able to stay suspended in the water column; a potential competitive advantage when resources are limiting. The other Chlorophycean species in my experiment, *Mougeotia*, floated in the water column and did reach high biomass density when grown alone. However, *Mougeotia* was never a competitive dominant when grown with one or more species of diatom, even in with-*Daphnia* polycultures where *Mougeotia* was the only inedible species. Observations of live algae under a microscope suggest that the diatoms, which tend to sink in the water column, frequently attached to filaments of *Mougeotia*. It is possible that this interaction between *Mougeotia* and diatoms resulted in the relative underyielding of *Mougeotia* in my experiment. Though my experimental units do not have the same complexity as a freshwater lake, variation in sinking rates and physical interactions among phytoplankton species are important components of phytoplankton competition in lakes in qualitatively similar ways [32].

Overall, my experiment shows that, given the patchy distribution of an upper ‘predator’ trophic level, ‘prey’ species richness can have a relatively weak direct effect on prey standing biomass. The direct effects of competitor richness are known to vary across communities, but it is also broadly generalized that experiments and theory tend to show positive effects of competitor richness [10,43]. To date, most theory and experiments that address the direct effects of competitor species richness either ignore or exclude the consumptive effects of upper trophic levels [31]. Studies that do include upper trophic levels show that the effects of prey species richness can vary [25–27] but this group of studies is substantially smaller and has not been as thoroughly synthesized [2,9]. Predators and herbivores are common in natural communities and it is possible that negative or roughly neutral effects of competitor richness on standing competitor biomass are quite common at local and regional scales when upper trophic levels are present. More experiments are needed to fully understand the direct effects of competitor species richness in ecosystems where predators and herbivores have strong consumptive effects on competitor communities. When such studies find weak or null effects of species richness and added challenge is distinguishing mechanistic null effects of species richness from limitations in statistical power to detect directional effects. Close consideration of the yields of individual species in monoculture and polyculture, like the analyses I show above, can help explain and generalize the mechanistic effects of prey richness in the presence of predators.

## Supporting Information

S1 Fig Polyculture Diversity and Evenness. Top panels show average Shannon’s Diversity (*H* ± standard error) in 3- and 5-species polyculture across sampling dates at the regional scale (A), in no-*Daphnia* patches (B), and in with-*Daphnia* patches (C). Bottom panels show average evenness (*J* ± standard error) in 3- and 5-species polyculture across sampling dates at the regional scale (D), in no-*Daphnia* patches (E), and in with-*Daphnia* patches (F).
(TIF)
