# Supplementary figures and images for: Effect of Phytoplankton Richness on Phytoplankton Biomass Is Weak Where the Distribution of Herbivores is Patchy
Source: PLoS One. 2016 May 19;11(5):e0156057. doi: 10.1371/journal.pone.0156057 (PMC4873172; doi:10.1371/journal.pone.0156057)

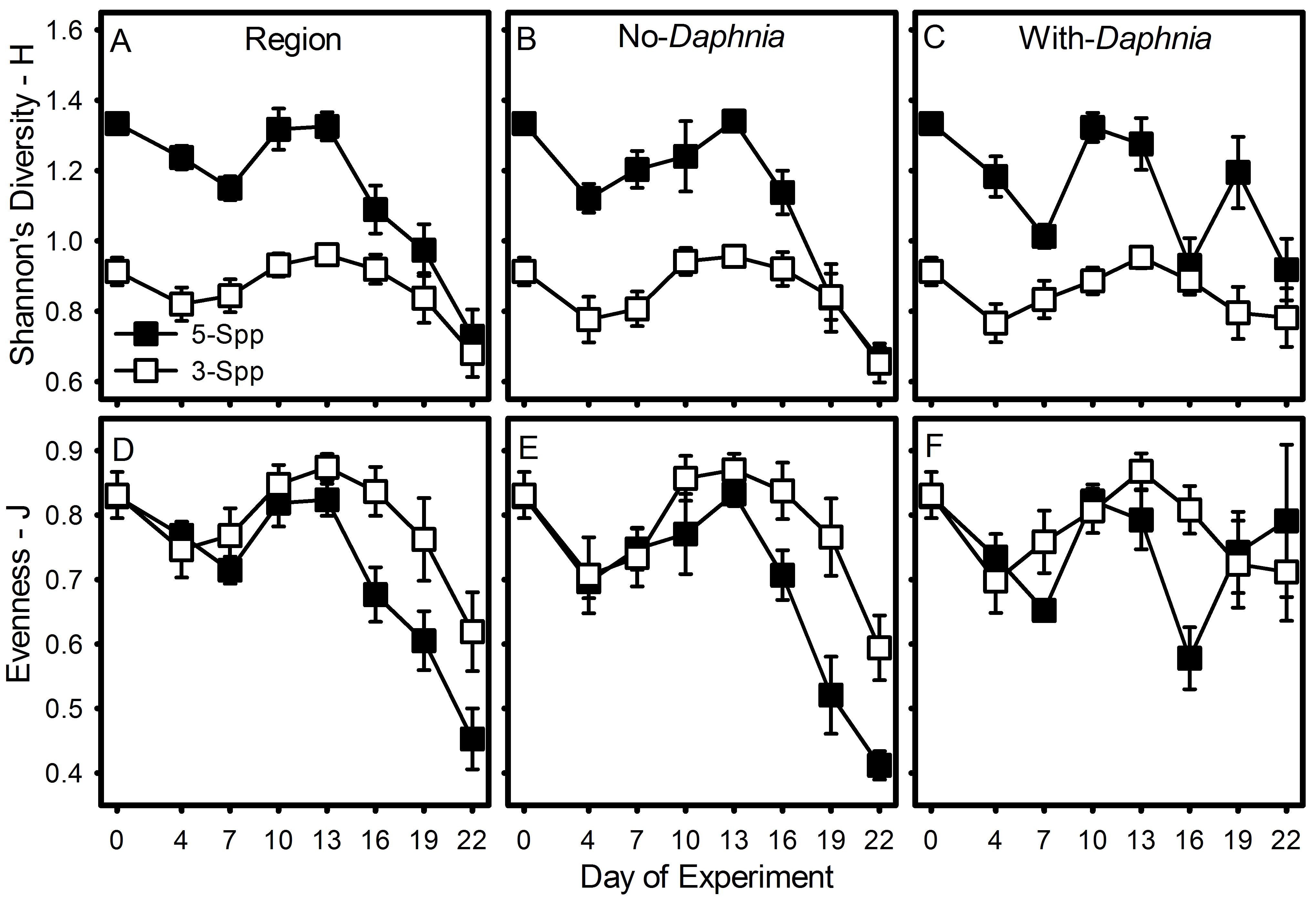

Supplement: S1 Fig — Top panels show average Shannon’s Diversity (H ± standard error) in 3- and 5-species polyculture across sampling dates at the regional scale (A), in no-Daphnia patches (B), and in with-Daphnia patches (C). Bottom panels show average evenness (J ± standard error) in 3- and 5-species polyculture across sampling dates at the regional scale (D), in no-Daphnia patches (E), and in with-Daphnia patches (F). (TIF) [file pone.0156057.s001.TIF]
